# Diet quality in late midlife is associated with faster walking speed in later life in women, but not men: findings from a prospective British birth cohort

**DOI:** 10.1017/S0007114519003313

**Published:** 2020-04-28

**Authors:** Thanasis G. Tektonidis, Shelly Coe, Patrick Esser, Jane Maddock, Sarah Buchanan, Foteini Mavrommati, Jonathan M. Schott, Hooshang Izadi, Marcus Richards, Helen Dawes

**Affiliations:** 1 Centre for Movement, Occupation and Rehabilitation Sciences, Faculty of Health and Life Sciences, Oxford Brookes University, Oxford OX3 0BP, UK; 2 Centre for Nutrition and Health, Faculty of Health and Life Sciences, Oxford Brookes University, Oxford OX3 0BP, UK; 3 CLOSER, Institute of Education, University College London, London WC1H 0NU, UK; 4 Dementia Research Centre, UCL Queen Square Institute of Neurology, University College London, London WCIN 3BG, UK; 5 Oxford University Hospitals NHS Foundation Trust, Research and Development, Joint Research Office, OUH Cowley, Oxford OX42PG, UK; 6 School of Engineering, Computing and Mathematics, Faculty of Technology, Design and Environment, Oxford Brookes University, Oxford OX33 1HX, UK; 7 MRC Unit for Lifelong Health and Ageing at UCL, University College London, London WC1B 5JU, UK; 8 Department of Neurology, Nuffield Department of Clinical Neurosciences, University of Oxford, Oxford OX3 9DU, UK

**Keywords:** Nutrition, Physical capability, Epidemiology, Healthy Eating Index

## Abstract

Healthy diet has been linked to better age-related functioning, but evidence on the relationship of diet quality in late midlife and measures of physical capability in later life is limited. Research on potential sex differences in this relationship is scarce. The aim was to investigate the prospective association between overall diet quality, as assessed by the Healthy Eating Index-2015 (HEI-2015) at 60–64 years and measures of walking speed 7 years later, among men and women from the Insight 46, a neuroscience sub-study of the Medical Research Council National Survey of Health and Development. Diet was assessed at 60–64 years using 5-d food diaries, from which total HEI-2015 was calculated. At 69–71 years, walking speed was estimated during four 10-m walks at self-selected pace, using inertial measurement units. Multivariable linear regression models with sex as a modifier, controlling for age, follow-up, lifestyle, health/social variables and physical performance, were used. The final sample consists of 164 women and 167 men (*n* 331). Women had higher HEI-2015 and slower walking speed than men. A 10-point increase in HEI-2015 was associated with faster walking speed among women (B 0·024, 95 % CI 0·006, 0·043), but not men. The association remained significant in the multivariable model (B 0·021, 95 % CI 0·003, 0·040). In women, higher diet quality in late midlife is associated with faster walking speed. A healthy diet in late midlife is likely to contribute towards better age-related physical capability, and sex differences are likely to affect this relationship.

Life expectancy is increasing in middle- and high-income countries, although this is typically accompanied by deterioration in physical and cognitive health^(1)^, with physical capacity decline accelerating up to 20 % per 10 years in 70-year-old people^(2)^. Rates of decline vary among individuals, and differences in trajectories of muscle wasting by sex are well observed^(3)^. Nonetheless, the musculoskeletal system is considered a good reflector of the rate of decline of physical function in later life^(4)^, a concept also referred to as physical capability^(5)^.

Diet is proposed to play an important role in slowing the progression of the decline in physical capability^(6)^. A healthy diet, as described by the WHO^(7)^, has been found to delay the rates of decline in physical capability by limiting skeletal muscle and bone mass loss^(8)^, reducing oxidative stress damage and excessive levels of inflammation^(9)^, as well as lowering the incidence of chronic and neurodegenerative diseases^(7)^.

To date, research on diet and physical capability and performance of older people has mainly focused on single nutrients (vitamin D, Ca, protein, carotenoids)^(10,11)^ or food items rich in antioxidants and anti-inflammatory factors, such as greens, vegetables and whole-grain foods^(12)^, which are important for musculoskeletal function and the prevention of sarcopenia^(13)^; yet nutrients are not consumed in isolation, and it is the synergistic effect of an overall healthy diet^(14)^ which protects against functional decline^(15,16)^. Healthy dietary patterns such as the Mediterranean and Nordic diets have shown an association of higher diet quality and measures of physical performance^(17–19)^; however, regional dietary patterns may not be appropriate in all contexts due to differences in food preference, availability and accessibility.

Instead, diet indexes such as the US-based Healthy Eating Index-2015 (HEI-2015)^(20)^ are likely to reflect diet quality of most Western populations, independently of specific food items and in line with the latest dietary guidelines^(21)^, which are comparable with those in the Eatwell Guide the UK^(22)^. HEI has been associated with lower disability rates and mortality in older people^(23)^. Emerging evidence on the relationship between high HEI scores at midlife and better physical performance^(19)^ as well as physical function^(16)^ has been published; yet findings were indicative of cross-sectional associations^(19)^, or measures of physical functioning were self-reported and sample included only women^(16)^.

Interestingly, findings from the National Survey of Health and Development (NSHD) suggest a positive association between data-derived adulthood diet quality and objective measures of physical performance in late midlife^(24)^, with diet at late midlife being of particular importance. But to further understand the impact of diet at this age, it would be useful to assess diet quality according to evidence-based healthy dietary recommendations. Finally, using an objective measure of physical capability that reflects cognitive functioning^(25)^, musculoskeletal changes in older people and predicts survival^(26)^ would also be of great importance.

Therefore, the purpose of the present study was to investigate the extent that overall diet quality at age 60–64 years, as indicated by an evidence-based diet quality index, is related to walking speed, which is an objective measure of physical capability, in later life of men and women from the longest running British birth cohort.

## Methods

### Design and study population

**Fig. 1.**
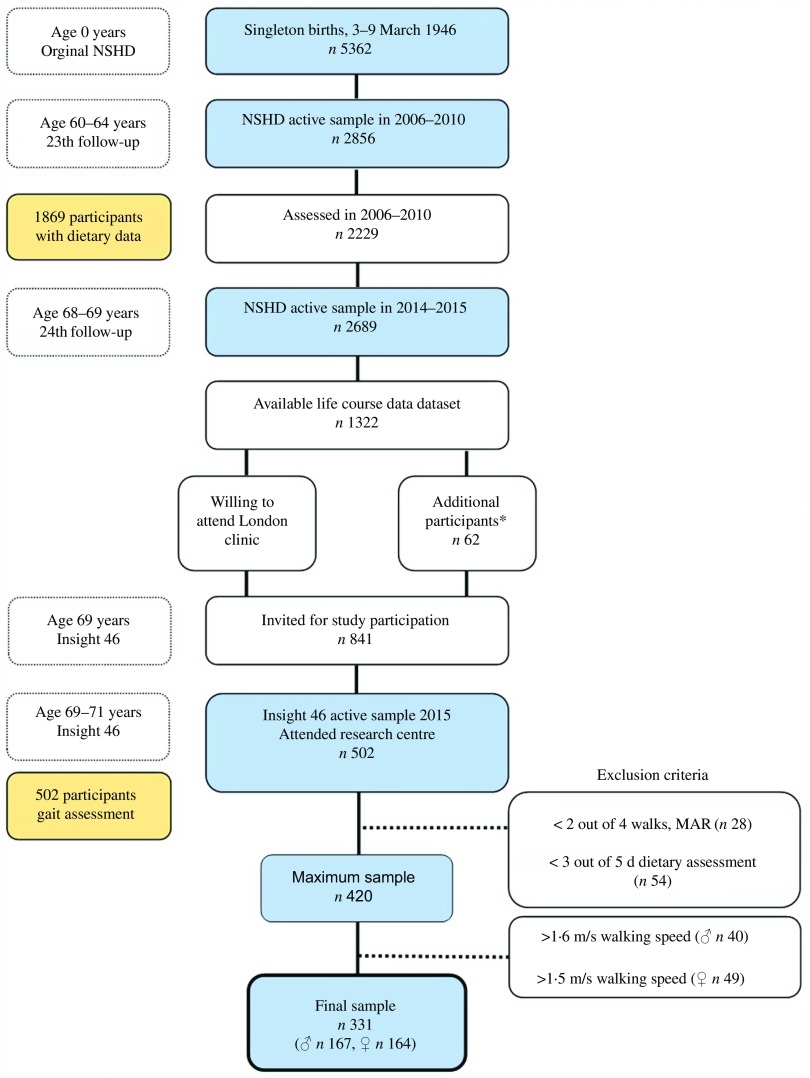
Number of participants in the National Survey of Health and Development (NSHD)/Insight 46 and selection criteria for the present study. MAR, missing at random. * To reach target sample of 500, participants without full set of life course data were included.

Insight 46 is a neuroscience sub-study of the Medical Research Council (MRC) NSHD, consisting of 502 participants of the original NSHD, who were active during the 24th follow-up in 2014–2015 (68–69 years, *n* 2689)^(27)^. The NSHD cohort has been described in detail elsewhere^(28)^. A detailed overview of sampling eligibility, data collection procedure and response rates in Insight 46 has been recently published^(27,29)^. In brief, of 841 participants who had participated in the 23rd follow-up (2006–2010), with a set of key life course data available and who were willing to attend a London-based clinic, 502 were randomly recruited and underwent cognitive function assessment, including gait assessment during the period 2015–2017 (Fig. 1)^(27,29)^. Participants with <3 d of dietary data (*n* 54) and <2 walks in gait assessment (*n* 28) were excluded from the analysis to achieve optimal data validity. Men with walking speed >1·6 m/s (*n* 40) and women with >1·5 m/s (*n* 49) were further excluded, representing non-feasible values, as defined by two standard errors of normative values of walking speed from the previous literature^(30)^, as well as unpublished results. The final sample for the present study consists of 331 people (167 men, 164 women) from the NSHD/Insight 46, aged 60–64 years in 2006–2010 (Fig. 1). The study was conducted according to the guidelines of the Declaration of Helsinki; ethical approval was obtained from the Greater Manchester and the Scotland Research Ethics Committees (NSHD) and the National Research Ethics Service Committee London (14/LO/1173, Insight 46). Written informed consent was obtained from all the participants.

### Dietary assessment

Dietary data were collected at age 60–64 years (2006–2010), via 5-d estimated diet diaries^(31)^. Participants were asked to record all food and beverages consumed at all occasions in consecutive days including three weekdays and two weekends. Prior to completion, participants were provided with extended guidance notes and portion size photographs. Diaries were coded and analysed by the Medical Research Council in-house software, Diet in Nutrient Out^(32)^, from where the ‘McCance and Widdowson’s’ food tables were used to estimate macro- and micronutrients, considering food composition, variation in food items and standardised portion sizes^(31)^. The food diaries have been validated^(33)^, and recommended energy cut-off points (<500, >3500 kcal (<2090, >14 640 kJ) for women, <800, >4000 kcal (<3350, >16 740 kJ) for men) were applied to account for misreporting^(34)^. No misreporters were identified.

### Healthy Eating Index-2015

The HEI-2015 is a multidimensional composite score, assessing overall diet quality according to the latest Dietary Guidelines for Americans (2015–2020)^(20)^. HEI-2015 consists of thirteen food and nutrient-based components, nine to be consumed in adequacy and four in moderation. An overview of the components and the scoring criteria are shown in online Supplementary Table S1^(20)^. In brief, all components are equally important and their score ranges from 0 to 10 points. Specific constructs of the diet, for example, fruit, vegetable and protein, are represented by two components (each ranging 0–5 points) to comply with the latest dietary guidelines^(20)^. The total score ranges from 0 to 100 points with higher values reflecting better diet quality. A maximum of 100 points reflects perfect adherence to the Dietary Guidelines for Americans.

**Fig. 2.**
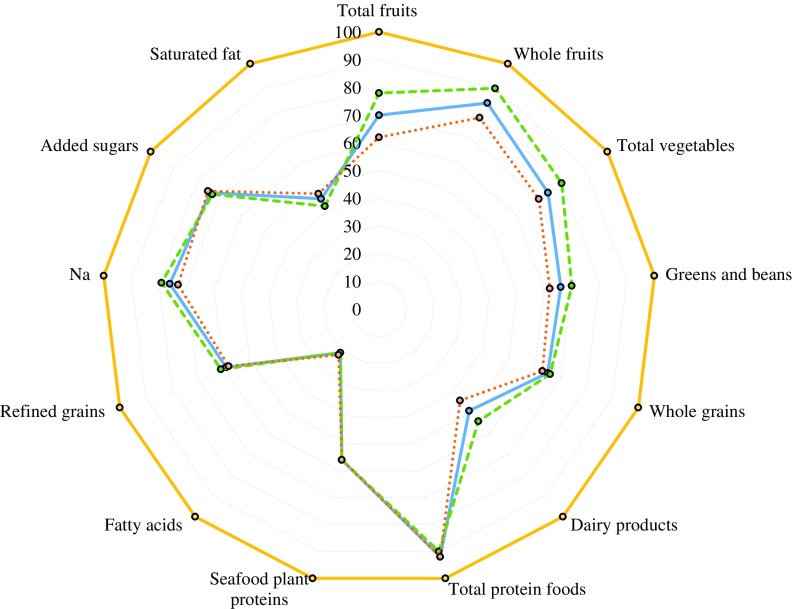
Radar plot of Healthy Eating Index-2015 (HEI-2015) components and total scores at age 60–64 years of participants with valid data of walking speed in single gait task at age 69–71 years, overall and by sex, National Survey of Health and Development (NSHD)/Insight 46, *n* 331. Percentage of maximum points received for each component on average, overall and by sex, with 0 % in the centre and 100 % at the outer edge. A perfect HEI-2015 scores a total maximum of 100 points (100 % in each component) and is represented by the dashed line around the perimeter of the graph. 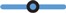, Total HEI-2015, mean: 62·0, overall, *n* 331; 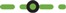, total HEI-2015, mean: 63·0, women, *n* 164; 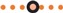, total HEI-2015, mean: 60·0, men, *n* 167; 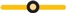, perfect HEI-2015 scoring max of 100·0 points.

Added sugars were estimated as total sugars minus natural sugars for all fruit, vegetables and dairy products, by using US^(35)^ and UK^(36)^ food databases. The US MyPyramid Equivalents Database^(37)^ and data from the National Diet and Nutrition Survey (UK)^(38)^ were also used, to select items for HEI components. HEI component and total scores were calculated according to scoring standards^(20)^. Units of scoring standards in HEI score were converted to g instead of ounces and cup equivalents. For optimal visualisation, radar plots were used to determine differences in patterns of diet quality between sexes by HEI-2015 component and total scores (Fig. 2).

### Walking speed

Walking speed was estimated during gait assessment in the clinic visit in 2015–2017 (69–71 years) over a pre-marked obstacle free walkway^(27)^. Participants were asked to perform four standard 10-m walks at self-selected walking pace, whilst being instrumented by an inertial measurement unit (Life Performance Research), attached over the fourth lumbar vertebra by a double adhesive tape^(39)^. Data were analysed using a bespoke programme (LabVIEW 15.1f, National Instruments). To account for anthropometric differences between sexes, which affect walking pattern, derived walking speed was normalised for leg length at age 69–71 years^(40)^.

### Covariates

Identification of descriptive parameters up to age 64 years was done both a priori as well as according to previous evidence in the NSHD^(6,24,31)^. Education attainment up to 26 years (no – compulsory – higher education), occupation at age 53 years (professional – skilled – unskilled) and the following characteristics at age 60–64 years were self-reported: marital status (married/with partner – no partner), leisure time physical activity during past 4 weeks (none – one to four times per week – five or more times per week), smoking status (former – current – never), use of at least one dietary supplement (yes – no). BMI was calculated as body weight in kg by height in m squared, both measured during the clinic/home visit at age 60–64 years. Total number of co-morbidities up to age 60–64 years was estimated as a construct of either self-reported or diagnosed prevalence or incidence or medication for each of the following conditions: CVD including angina, heart failure, myocardial infarction and coronary artery bypass graft, diabetes type 1 or 2, stroke, cancer, hypertension and hypercholesterolaemia. Balance ability was measured as the longest time, to a maximum of 30 s, for which participants could maintain a one-legged stance in a standard position with eyes open. Missing values of all variables were <4 % of study sample, with the exception of BMI equal to 10 % (*n* 331).

### Statistical methods

Descriptive characteristics were presented as mean values and standard deviations for continuous (independent samples *t* tests) and frequencies for categorical variables (*χ* ^2^ tests), overall and by sex. Unadjusted, sex-adjusted and multivariable linear regression models were used to determine the association between 10-point increment of HEI-2015 and walking speed. Due to evidence from previous literature^(18,41)^, a moderating effect of sex was explored and detected and further analysis was stratified by sex. A 10-point increment in HEI-2015 was selected to reflect more meaningful changes in walking speed. A *post hoc* estimator was used to detect a medium effect size on up to twenty independent predictors, on an *α* level of 0·05 and statistical power of 0·8 (*n* estimated = 210 *v*. *n* study = 331)^(42)^. The multivariable models were adjusted for age at dietary assessment, follow-up period, occupation, education, marital status, leisure physical activity, smoking, supplement use, total number of co-morbidities, BMI and balance time. In sensitivity analyses, the relationship between the thirteen individual HEI-2015 component scores and walking speed was also explored in women using simple and multivariable linear regression models with stepwise function and same covariate adjustments. All tests were performed using SPSS version 25 (IBM Co.). Statistical significance was set at *P* ≤ 0·05 (two-sided) for all tests.

## Results

Characteristics of participants up to age 60–64 years are presented in Table 1. On average, women were less likely to have professional occupation, less likely to be married and performed worse in the balance test than men; yet they were more moderately active, used supplements more and had higher diet quality (Fig. 2 and Table 2). Men had faster walking speeds than women at age 69–71 years (Table 3).

**Table 1.** Descriptive characteristics of participants up to age 60–64 years with valid data of walking speed in single gait task at age 69–71 years, overall and by sex, National Survey of Health and Development (NSHD)/Insight 46, *n* 331 (Numbers and percentages; mean values and standard deviations)

	Overall (*n* 331)			Women (*n* 164)			Men (*n* 1670		
	*n*		%	*n*		%	*n*		%
Age, 2006–2010 (years)									
Mean		63·3			63·3			63·3	
sd		1·1			1·0			1·1	
Occupation									
Professional	212·0		64·0	90·0		55·0	122·0		73·0†
Skilled	117·0		35·0	74·0		45·0*	43·0		26·0
Unskilled	2·0		1·0	0·0		0·0	2·0		1·0
≥Secondary education	250·0		76·0	123·0		75·0	127·0		76·0
Married/with partner	268·0		81·0	126·0		77·0	142·0		85·0‡
Total co-morbidities (*n* > 2)	36·0		12·0	16·0		10·0	20·0		12·0
Leisure physical activity									
None	173·0		52·0	79·0		48·0	94·0		56·0
1–4 times/week	66·0		20·0	41·0		25·0‡	25·0		15·0
≥5 times/week	92·0		28·0	44·0		27·0	48·0		29·0
Smoking status (current)	14·0		4·0	7·0		4·0	7·0		4·0
Supplement use (yes)	159·0		48·0	92·0		56·0†	67·0		40·0
BMI (kg/m^2^)									
Mean		27·1			27·1			27·1	
sd		3·8			4·2			3·2	
Balance time, eyes open, ≥30 s§	179·0		54·0	75·0		46·0	104·0		62·0†

* *P* ≤ 0·001.

† *P* ≤ 0·01.

‡ *P* ≤ 0·05.

§ Balance time is presented as percentage of participants who achieved balance of 30 s with eyes open.

**Table 2.** The Healthy Eating Index-2015 (HEI-2015) total and component scores of participants at age 60–64 years with valid walking speed at age 69–71 years, overall and by sex, National Survey of Health and Development (NSHD)/Insight 46, *n* 331 (Median values and ranges; mean values and standard deviations; numbers and percentages)

	Overall (*n* 331)				Women (*n* 164)				Men (*n* 167)			
	Median		Range		Median		Range		Median		Range	
HEI-2015, 0·0–100·0	61·0		27·0–93·0		63·0		27·0–93·0		59·0		30·0–92·0	
Component scores	Mean	sd	*n* max score‡	%	Mean	sd	*n* max score‡	%	Mean	sd	*n* max score‡	%
Adequacy												
Total fruits§	3·5	1·6	140·0	42·0	3·9	1·4*	85·0	52·0*	3·1	1·7	55·0	33·0
Whole fruits§	4·2	1·4	243·0	73·0	4·5	1·1*	135·0	82·0*	3·9	1·6	108·0	65·0
Total vegetables§	3·7	1·1	121·0	36·0	4·0	1·0*	73·0	45·0*	3·5	1·1	48·0	29·0
Greens and beans§	3·3	1·7	123·0	37·0	3·5	1·6	67·0	41·0†	3·1	1·7	56·0	34·0
Whole grains	6·5	3·5	119·0	36·0	6·6	3·4	63·0	38·0	6·3	3·6	56·0	34·0
Dairy products	4·9	2·3	17·0	5·0	5·4	2·5*	14·0	9·0†	4·4	2·0	3·0	2·0
Total protein foods§	4·6	0·8	252·0	76·0	4·5	0·9	118·0	72·0	4·6	0·7†	134·0	80·0
Seafood and plant proteins§	2·8	1·9	109·0	33·0	2·8	1·9	56·0	34·0	2·8	1·9	53·0	32·0
Fatty acids	2·1	2·4	6·0	2·0	2·1	2·6	5·0	3·0	2·2	2·2	1·0	1·0
Moderation												
Refined grains	5·9	3·4	86·0	26·0	6·1	3·3	47·0	29·0	5·8	3·5	39·0	23·0
Na	7·6	2·5	115·0	35·0	7·9	2·5†	68·0	42·0†	7·3	2·5	47·0	28·0
Added sugars	7·4	2·0	56·0	17·0	7·3	2·0	29·0	18·0	7·5	1·9	27·0	16·0
Saturated fat	4·5	3·0	20·0	6·0	4·2	3·0	10·0	6·0	4·7	3·0	10·0	6·0

* *P* ≤ 0·001.

† *P* ≤ 0·05.

‡ Number of participants who met the standard for maximum score in each component.

§ Maximum score is 5; for the rest of the components, the maximum score is 10.

**Table 3.** Walking speed in single gait task at age 69–71 years by 10-point increment of the Healthy Eating Index-2015 (HEI-2015) score of participants at age 60–64 years, stratified by sex, National Survey of Health and Development (NSHD)/Insight 46, *n* 331 (Unstandardised coefficients (B) and 95 % confidence intervals; mean values and standard deviations)

	HEI-2015, 0·0–100·0, per 10-point increment					
	Women (*n* 164)			Men (*n* 167)		
	B		95 % CI	B		95 % CI
Walking speed (m/s)						
Mean		1·32			1·39	
sd		0·14			0·15	
Sex stratified‡	0·024		0·006, 0·043*	–0·012		–0·030, 0·006
Multivariable adjusted§	0·021		0·003, 0·040†	–0·009		–0·028, 0·009
Normalised walking speed (m/s)						
Mean		0·44			0·47	
sd		0·05			0·05	
Sex stratified	0·008		0·002, 0·014†	–0·004		–0·010, 0·002
Multivariable adjusted‖	0·008		0·001, 0·014†	–0·005		–0·011, 0·001

* *P* ≤ 0·001.

† *P* ≤ 0·05.

‡ Sex stratified model: 10-point increment of HEI-2015 as predictor of walking speed, stratified by sex.

§ Adjusted for 2016–2010 factors: age, time period until gait, occupation, married/with partner, at least secondary education, smoking status, leisure physical activity per month, supplement use, number of co-morbidities, BMI and balance time.

‖ Not adjusted for BMI because walking speed was already normalised for leg length.

Table 3 shows the associations of HEI-2015 and walking speed by sex. Over a median follow-up period of 7·2 years, no overall association was found; yet there was evidence for a moderating effect of sex. In stratified analysis, a 10-point increment in HEI-2015 was associated with faster walking speed among women but not men. This association remained following adjustment for confounders. No association was found for men in any model.

Of thirteen HEI-2015 components, higher scores for greens and beans, whole-grains and seafood, and plant proteins were associated with faster walking speed in women in the unadjusted model but only greens and beans and total protein foods in the multivariable model (online Supplementary Table S2).

## Discussion

Utilising data from the longest running British birth cohort, the present study showed that diet quality, as indicated by higher HEI-2015 scores at age 60–64 years, was associated with faster walking speed among women, but not men, 7 years later, independently of a wide range of factors. To the best of our knowledge, this is the first prospective study to show sex differences in the relationship between a valid diet quality index in late midlife that reflects current dietary guidelines and better physical capability in later life, as indicated by walking speed. It further confirms evidence that healthy dietary choices at late midlife, as those reflected by high HEI-2015 scores, may slow down the rate of decline of physical capability in later life, as indicated by faster walking.

It is novel in the present study that high diet quality, as indicated by high HEI-2015 scores, was linked to faster walking speed in older women but not men. Similar sex differences have been observed in studies investigating associations between diet and physical function^(11,18,41)^. A healthy Nordic diet has been associated with better physical performance, measured by the Senior Fitness Test^(18)^ and with muscle strength^(41)^, only in women. Data from the UK Hertfordshire Cohort Study revealed similar sex difference in the association between micronutrients and vitamins with physical performance, with shorter 3-m walk times among older women with higher intakes of antioxidants, vitamin D and energy, but not men^(11)^. Potential explanations of these findings can be hypothesised. First, women had higher diet quality compared with men in the present study and higher scores in assumedly beneficial food groups. Likewise, women of similar age in the Multiethnic Cohort (USA) had higher HEI-2015 scores than men^(23)^. HEI-2015 is a multidimensional score which allows for different combinations of components to achieve the same total score^(20)^. Several studies have shown sex differences in the general direction of healthier food choices for women than men with regard to both food groups and nutrient intakes^(31,43,44)^. Results from the NSHD show that women in the present study have increased intakes of antioxidants overtime due to higher consumption of fruit and vegetables and lower consumption of whole milk, butter and red meat, compared with men^(31,43,44)^. We should consider the possibility of biological sex differences in ageing, with men having more muscle and bone mass than women over the lifespan, despite decline rates being much faster for men in older age^(3)^. Sex hormones are well known to decline much steeper in older women than men, resulting in significant loss of physical function^(45)^. Despite women having on average longer life expectancy than men, they also have higher morbidity rates^(1)^, weaker musculoskeletal system^(46)^ and slower walking speed values in the study. Therefore, given considerable evidence that links healthy diet to muscle mass, strength and function of older adults^(8)^, it is likely to be more important for the more ‘vulnerable’ women to maintain high diet quality than men, who tend to be more robust despite lower life expectancy. This may also indicate a threshold in walking speed below which variability in diet quality might be of greater importance. Finally, sex differences in the observed association may reflect differences in long-term cumulative exposure of high diet quality over the lifespan, which are linked to physical performance in later life, as shown previously^(6,24)^.

Evidence has shown an association between higher diet quality and healthy ageing^(16)^, overall health-related quality of life^(47)^ and better physical performance among older adults^(19)^. A recent analysis from the Nurses’ Health Study showed a lower risk of self-reported physical impairment among participants with higher alternative HEI-2010 scores over an 18-year period among older adults^(48)^. In our study, a 37-unit increase in HEI-2015 was associated with a considerable change of 0·1 m/s in walking speed, proposed to be predictive of survival among older people^(26)^, thus suggesting a small but important estimate. Similarly, after controlling for the same kind of potential confounders in the present study, a cross-sectional association between higher HEI-2005 total scores and faster walking speed among approximately 2100 older men and women was found in the USA; yet compared with the present study, the sample was older and was less high-functioning, as indicated by slower walking speed, higher prevalence of co-morbidities and higher BMI^(19)^.

Using healthy dietary patterns, albeit cultural- and regional-specific, cross-sectional and observational studies support the finding of the present study regarding the relationship of high diet quality in midlife with better physical function in later life^(15,17,18,24)^. In a prospective study of community-dwelling older adults, walking speed over an 8-year period was faster among those with better diet quality as indicated by higher MedDiet scores at baseline, suggesting a long-term effect of diet on mobility performance^(17)^. High diet quality, as reflected by a healthy Nordic diet score, was associated with better physical performance in the 6-min walk test among Nordic women over 60 years over a 10-year period^(18)^. Despite cultural and regional differences in food choice, all these dietary patterns highlight the importance of diet quality over quantity with the main focus on intakes of plant foods, whole grains and fish/long-chain *n*-3 PUFA as well as lower intakes of red and processed meats, added sugars and saturated fat^(17,18)^. Therefore, it is likely that overall diet quality, rather than a specific diet, is important for maintaining physical capability, as shown in the present study.

The present study also showed that higher HEI-2015 scores for greens and beans, whole grains, total protein, seafood and plant proteins were associated with faster walking speed among women. Findings are consistent with evidence from a longitudinal study which showed that lower fruit and vegetable consumption among older people was associated with functional limitations and disability over a 17-year period^(12)^. The NSHD study assessed the effect of adult diet quality over 30 years on physical performance, as measured by chair rise, timed-up-and-go and standing balance tests and showed a positive association between early adulthood and early older age dietary patterns, high in fruits, vegetables and whole grains, and measures of physical performance^(24)^. It is interesting that using data from the same cohort, the present study confirmed those findings additionally for walking speed, which is another valid measure of physical capability. Regarding protein, our findings support previous research from the NSHD, which suggested a weak relationship of higher protein intake in lifetime adulthood with better physical capability in older age^(6)^; the low strength of the association might be due to the study assessing quantity as total protein intake rather than quality as source of protein (seafood *v*. meat protein). Finally, the UK Hertfordshire Cohort Study further confirms our results for seafood and plant protein foods, as they also showed a positive association of fatty fish consumption, rich in vitamin D and *n*-3 fatty acids and objective measures of physical capability among approximately 2000 older adults^(49)^.

The study has major strengths including the longest running and in most cases nationally representative British birth cohort of women and men, long follow-up to detect relatively long-term dietary effects, the use of a valid measure of diet quality^(20)^, objective measures of walking speed indicating physical capability^(50)^ and detailed information on a broad range of covariates at baseline. Dietary assessment was undertaken using food diaries over all seasons, which despite potential for measurement error, are still considered the ‘gold standard’ method^(51)^ and provide extensive information about food type and thus reflect diet quality. Finally, all study participants were within the same age group and we also controlled for age at dietary assessment, hence limiting important source of confounding.

Limitations include participants being more likely to have provided dietary information at age 60–64 years than those in the original NSHD but not in Insight 46; hence, they were likely more health conscious^(29)^. Interim health events between baseline and follow-up were not available; however, the models were controlled for number of co-morbidities at baseline. In addition, participants in the Insight 46 were in general healthier individuals^(29)^. Indeed, walking speed of men and women in the present study was significantly higher than normative values^(52)^, indicating overall healthy ageing. Walking speed via inertial measurement unit was not assessed at baseline, and this might have affected the observed associations; however, findings likely express true relationship as models were controlled for physical activity and balance tests at baseline and previous literature of similar design supports the present findings^(18,41)^. Moreover, men tend to outperform women in walking status at all ages^(53)^, conditional on anthropometric differences^(52)^ with similar trends in speed decline by sex^(53)^. Therefore, dramatic changes of speed between baseline and follow-up by sex are unlikely. Although selection procedure of the Insight 46 was thoroughly designed, potential collider bias deriving may lead to biased estimates of the observed associations^(54)^. Attrition rates and loss to follow-ups are common issues in longitudinal studies; however, the cohort in 2006 was still representative of the British population in most aspects^(29)^. Only white British-born people were included; thus generalisability should be made with caution; however, when compared with the National Diet and Nutrition Survey, reflecting dietary intake of the British population, the original NSHD sample showed notable agreement regarding age-matched trends in dietary intake^(31,38,44)^. Despite the prospective design and thorough adjustment for major confounders, we cannot exclude the possibility of residual confounding. Although the sample size was relatively small, it is adequate to detect the observed associations, as per *post hoc* analysis^(42)^.

In conclusion, the present study adds evidence for the relationship of high diet quality in late midlife, in particular among women, and better physical capability in later life, as indicated by faster walking speed, reflecting healthier ageing. Despite the estimate size being relatively small, it is important that adaptation to high diet quality at midlife, for example, increase in consumption of greens, whole grains and whole fruits alongside lower intakes of added sugars and saturated fat from animal sources, in line with established dietary guidelines, is likely to contribute towards better physical capability in later life and sex differences are likely to affect this relationship, suggesting different strategies in lifestyle interventions of ageing people to be further explored.

## Supplementary material

For supplementary material accompanying this paper visit http://dx.doi.org/10.1017/S0007114519003313. click here to view supplementary material
