# Supplementary material for: Diet quality in late midlife is associated with faster walking speed in later life in women, but not men: findings from a prospective British birth cohort
Source: Br J Nutr. 2019 Dec 16;123(8):913–21. doi: 10.1017/S0007114519003313 (PMC7056405; doi:10.1017/S0007114519003313)
Supplement: Supplementary file 1 [file S0007114519003313sup001.docx]

**Supplementary Material:**

**Supplementary Table S 1.** Original Healthy Eating Index-2015 (HEI-2015) components & scoring system a using standardized grams equivalents from the MyPyramid Equivalents Database.

**Supplementary Table S 2.** Unstandardized coefficients (B) and 95% confidence intervals 95% CIs of walking speed in single gait task at age 69-71 by component scores of the Healthy Eating Index-2015 of women at age 60-64, NSHD/Insight 46

| Supplementary Table S 1. Original Healthy Eating Index-2015 (HEI-2015) components & scoring system a using standardized grams equivalents from the MyPyramid Equivalents Database. | | | |
| --- | --- | --- | --- |
| **Component** | **Maximum Points** | **Standard for max score** | **Standard for min score (zero)** |
| **Adequacy** |  |  |  |
| Total Fruits ^b^ | 5 | ≥ ~170 grams ^i^ equiv. per 1,000 kcal | No Fruit |
| Whole Fruits ^c^ | 5 | ≥ ~150 grams equiv. per 1,000 kcal | No Whole Fruit |
| Total Vegetables ^d^ | 5 | ≥ ~160 grams equiv. per 1,000 kcal | No Vegetables |
| Greens & Beans ^d^ | 5 | ≥ ~125 grams equiv. per 1,000 kcal | No Dark Green Vegetables or Legumes |
| Whole Grains | 10 | ≥ ~45 grams equiv. per 1,000 kcal | No Whole Grains |
| Dairy ^e^ | 10 | ≥ ~320 grams equiv. per 1,000 kcal | No Dairy |
| Total Protein Foods ^f^ | 5 | ≥ ~75 grams equiv. per 1,000 kcal | No Protein Foods |
| Seafood & Plant Proteins ^f, g^ | 5 | ≥ ~25 grams equiv. per 1,000 kcal | No Seafood or Plant Proteins |
| Fatty Acids ^h^ | 10 | (PUFAs + MUFAs)/SFAs ≥2.5 | (PUFAs + MUFAs)/SFAs ≤1.2 |
| **Moderation** |  |  |  |
| Refined Grains | 10 | ~55 grams equiv. per 1,000 kcal | ≥ ~130 grams equiv. per 1,000 kcal |
| Sodium | 10 | ≤1.1 gram per 1,000 kcal | ≥2.0 grams per 1,000 kcal |
| Added Sugars | 10 | ≤6.5% of energy intake | ≥26% of energy intake |
| Saturated Fat | 10 | ≤8% of energy intake | ≥16% of energy intake |
| ^a^ Intakes between the minimum and maximum standards are scored proportionately, ^b^ Includes 100% fruit juice, ^c^ Includes all forms except juice, ^d^ Includes legumes (beans and peas), ^e^ Includes all milk products, such as fluid milk, yogurt, and cheese, and fortified soy beverages, ^f^ Includes legumes (beans and peas), ^g^ Includes seafood, nuts, seeds, soy products (other than beverages), and legumes (beans and peas), ^h^ Ratio of poly- and monounsaturated fatty acids (PUFAs and MUFAs) to saturated fatty acids (SFAs), ^I^ Grams equivalents are the average values of grams of all food items comprising each component. | | | |

| Supplementary Table S 2. Predicted values of walking speed in single gait task at age 69-71y by component scores of the Healthy Eating Index-2015 of women at age 60-64y, NSHD/Insight 46 | | | | | | | | |
| --- | --- | --- | --- | --- | --- | --- | --- | --- |
|  | **Walking speed, m/s** | | | | **Normalized walking speed, -** | | | |
| **HEI-2015 component scores** | **Unadjusted** | | **Multivariable adjusted ^a^** | | **Unadjusted** | | **Multivariable adjusted ^a^** | |
| **Adequacy** |  | |  | |  | |  | |
|  | B | 95% CI | B | 95% CI | B | 95% CI | B | 95% CI |
| Total Fruits | 0.010 | -0.130, .0151 | 0.003 | -0.161, 0.167 | 0.011 | -0.127, 0.149 | 0.005 | -0.144, 0.154 |
| Whole Fruits | 0.048 | -0.085, 0.182 | -0.010 | -0.163, 0.143 | 0.049 | -0.086, 0.184 | 0.015 | -0.134, 0.164 |
| Total Vegetables | -0.006 | -0.147, 0.135 | 0.026 | -0.124, 0.176 | -0.007 | -0.160, 0.146 | -0.001 | -0.151, 0.151 |
| Greens & Beans | 0.020 | 0.004, 0.035^†^ | 0.018 | 0.002, 0.034^†^ | 0.006 | 0.001, 0.012^*^ | 0.006 | 0.001, 0.012^†^ |
| Whole Grains | 0.008 | 0.001, 0.015^†^ | 0.079 | -0.059, 0.217 | 0.002 | 0.001, 0.005^†^ | 0.088 | -0.026, 0.202 |
| Dairy | -0.035 | -0.170, 0.100 | -0.031 | -0.148, 0.086 | -0.037 | -0.173, 0.099 | -0.030 | -0.169, 0.109 |
| Total Protein Foods | 0.063 | -0.072, 0.200 | 0.031 | 0.001, 0.061^†^ | 0.061 | -0.075, 0.197 | 0.010 | 0.001, 0.020^†^ |
| Seafood & Plant Proteins | 0.014 | 0.001, 0.026^†^ | 0.072 | -0.070, 0.214 | 0.005 | 0.001, 0.009^†^ | 0.080 | -0.061, 0.221 |
| Fatty Acids | -0.016 | -0.148, 0.116 | 0.038 | -0.104, 0.180 | -0.018 | -0.151, 0.116 | 0.040 | -0.099, 0.179 |
| **Moderation** |  | |  | |  | |  | |
| Refined Grains | -0.011 | -0.129, 0.130 | 0.008 | -0.131, 0.147 | -0.015 | -0.200, 0.170 | 0.003 | -0.123, 0.129 |
| Sodium | 0.075 | -0.056, 0.206 | 0.055 | -0.082, 0.192 | 0.074 | -0.057, 0.205 | 0.067 | -0.067, 0.201 |
| Added Sugars | -0.047 | -0.182, 0.088 | 0.024 | -0.117, 0.165 | -0.047 | -0.183, 0.088 | 0.013 | -0.137, 0.163 |
| Saturated Fat | 0.028 | -0.114, 0.170 | 0.065 | -0.073, 0.203 | 0.026 | -0.114, 0.166 | 0.073 | -0.063, 0.209 |
| NSHD, National Survey of Health and Development, HEI-2015, Healthy Eating Index-2015, B, unstandardized coefficient, 95% CI, 95% confidence interval  ^a^ Adjustments are as per main analysis.  ^*^ p value ≤ .01, ^†^  p value ≤ .05 | | | | | | | | |
